# A New Protocol for Molecular Detection of *Cyclospora cayetanensis* as Contaminants of Berry Fruits

**DOI:** 10.3389/fmicb.2019.01939

**Published:** 2019-08-27

**Authors:** Tamirat T. Temesgen, Kristoffer R. Tysnes, Lucy J. Robertson

**Affiliations:** Laboratory of Parasitology, Department of Food Safety and Infection Biology, Faculty of Veterinary Medicine, Norwegian University of Life Sciences, Oslo, Norway

**Keywords:** *Cyclospora cayetanensis*, berries, TaqMan probe, internal transcribed spacer-1, method development, duplex qPCR, contamination, detection

## Abstract

*Cyclospora cayetanensis* is a coccidian parasite that is associated with foodborne outbreaks of gastrointestinal illnesses. Raspberries have been implicated as a vehicle of infection in some of these outbreaks. Most of the molecular techniques used for the detection of parasites commonly use the 18s rRNA as a target gene, which is highly conserved. The conserved nature of the 18s rRNA gene among coccidia means that there is potential for cross-reactivity from primers intended to target this gene in *C. cayetanensis* with the same gene in related coccidia. This provides an additional challenge in developing a specific detection method. The aim of this study is to develop a new, more specific assay to detect *C. cayetanensis* in berry fruits. This new assay, targeting the internal transcribed spacer 1 (ITS-1) region, was tested on three different berry matrices: raspberries, blueberries, and strawberries. The new assay showed good efficiency (102%), linearity (*r*^2^ = 0.999), repeatability (standard deviation of C_q_ 0.2 (95% CI: 0.2, 0.3) and specificity for *Cyclospora*, with no cross-reactivity with related coccidia (*Toxoplasma gondii, Eimeria mitis, Cystoisospora canis*, and *Cryptosporidium parvum*) when tested *in vitro*. The method development was initially conducted using *Cyclospora* DNA only. After it was confirmed to have an acceptable performance, the method was evaluated using the oocysts of *C. cayetanensis*. The method was also improved by incorporating an internal control as a duplex in order to monitor PCR inhibition due to sample matrix components. The duplex assay also showed a good efficiency (100%) and linearity (*r*^2^ = 0.99). The results showed that the new assay has potential for standard use in food testing laboratories. Furthermore, results regarding important factors related to assay robustness are discussed.

## Introduction

*Cyclospora cayetanensis* is a coccidian parasite that has been associated with extensive foodborne outbreaks of gastrointestinal disease. The symptoms of cyclosporiasis include watery diarrhoea, nausea, loss of appetite, cramping, bloating, increased gas, weight loss, fatigue, and, less commonly, vomiting and low-grade fever. There have been frequent outbreaks of cyclosporiasis in the United States, with hundreds of people affected every year.

According to Centers for Disease Control and Prevention (CDC), 1065 laboratory-confirmed cases of cyclosporiasis from 40 states were recorded in spring/summer outbreaks in 2017, 384 laboratory-confirmed cases in 2016, 546 in 2015, and 304 in 2014^1^. However, the number of cases showed a dramatic increase in 2018; as of 1 October, 2018, 2,299 laboratory-confirmed cases of cyclosporiasis had been reported from 33 states. Epidemiological investigations indicated that some of the cases were linked to prepackaged vegetable trays sold at a convenience store chain and salads sold at a fast-food chain (Casillas et al., 2018). Previous outbreaks of cyclosporiasis have been commonly associated with imported contaminated raspberries, cilantro, basil, mesclun lettuce, and snow peas (Chacin-Bonilla, 2017).

As the number of parasites contaminating fresh produce is often likely to be low and there are difficulties in obtaining clean sample eluates, detection methods based on light microscopy are probably hampered by low sensitivity. Given that the sensitivity of molecular techniques, such as polymerase chain reaction (PCR), is often considerably higher than that of microscopy techniques, there have been considerable efforts directed towards development and validation of new protocols for detecting *C. cayetanensis* (Shields et al., 2013; Murphy et al., 2017, 2018; Almeria et al., 2018).

Fresh produce may be contaminated by oocysts from a variety of coccidian parasites (e.g., *Eimeria* spp., *Cryptosporidium* spp., and *Toxoplasma gondii*), and although there are many factors that can influence the likelihood of fresh produce being contaminated with different coccidian parasites, the level of specificity of any detection method is crucial. Molecular detection methods often target conserved regions of the genome, e.g., 18s rRNA, that are found in multiple copies. On the one hand, the multi-copy features of these loci are beneficial for assay sensitivity. On the other hand, because these are conserved regions they are very similar across closely related species. The latter feature makes it challenging to develop primers and probes that are specific for the target parasite and do not amplify corresponding genes in related parasites.

The more closely related non-target species are to a target species, the more likely that primers or probes may bind. Thus, although *C. cayetanensis* seems to infect only humans, there are several other species of *Cyclospora* that infect other animal hosts (including cattle, primates, and reptiles) (Lainson, 2005). These species are not infectious to humans but, if contaminating fresh produce, may be amplified due to their sequence similarity to *C. cayetanensis*. Less closely related species, such as coccidia in the genera *Cystoisospora, Eimeria*, and *Toxoplasma*, or even *Cryptosporidium*, may also be amplified by primers intended for *C. cayetanensis*, particularly if the target gene is highly conserved.

In our laboratory, cross-reactivity with the DNA from *T. gondii* was observed using the primers and probe described in the method currently used by the FDA for detection of *C. cayetanensis* from fresh produce (Murphy et al., 2017), albeit that in our laboratory the probe was labelled differently and had a different quencher. In addition, we found that the same primers and probe with the unmodified PCR conditions (Verweij et al., 2003) also cross-reacted with DNA from *Eimeria mitis* and *T. gondii*.

Thus, although the modification used in our laboratory was not identical with regards to the labelling and quenching, omission of the internal quencher would not affect the probe’s specificity because the internal quencher decreases background fluorescence, and hence increases the sensitivity and precision of the assay; this effect is significant for probes longer than 30 bp^2^.

Furthermore, given the increasing modifications and advances in PCR technology, it is important that assays designed for diagnostic testing are sufficiently robust that the principal components can be applied successfully with similar specificity and sensitivity despite minor alterations in, for example, primer and probe concentrations, annealing temperature, etc.

The internal transcribed spacer region of the genome, due to its non-coding nature, has a high degree of inter-species variation. The ITS-1 region of *C. cayetanensis* was shown to have a variation within and between samples collected from different geographical locations (Olivier et al., 2001). Another study showed that the high variability in the ITS-1 region of *C. cayetanensis* was intragenomic (Riner et al., 2010). This implies that the sensitivity of a method targeting the ITS-1 gene might be lower than one targeting the 18s-rRNA because the number of ITS-1 copies that matches a set of primers and probes can vary between oocysts.

Another molecular method using primers targeting the ITS-2 region of the *C. cayetanensis* genome was developed by Lalonde and Gajadhar (2008). As this method is based on conventional PCR it is both more time consuming and potentially less specific than methods based on TaqMan probe qPCR.

A further challenge when working with environmental samples, such as berries, is the presence of inhibitors in the sample matrices. It is known that berry fruits contain PCR inhibitors such as polysaccharides (e.g., pectin) and polyphenols (Schrader et al., 2012). This has been observed in the method developed for the detection of *C. cayetanensis* from cilantro and fresh raspberries (Murphy et al., 2017), where it was reported that inhibition due to matrix factors could result in complete absence of amplification. Significant inhibition, but not leading to complete absence of amplification might also occur and could be monitored by including an internal control as a duplex assay (Murphy et al., 2017). Although the inhibition could be reduced by fourfold dilution of the template, it must be borne in mind that diluting the template might also result in false-negative results, particularly when the concentration of the target DNA is very low. This means that using a DNA isolation protocol that counteracts PCR inhibitors would be preferable to diluting the template. The protocol developed by Murphy et al. (2017) has been approved by the United States. Food and Drug Administration (FDA) and is currently used for regulatory purposes (Almeria et al., 2018).

In the present study, we aimed to develop and evaluate a new protocol for molecular detection of *C. cayetanensis* from berry fruits, with emphasis on specificity, sensitivity and assay robustness, using the ITS-1 region as a target. We also incorporated an internal control, in a duplex method, to enable monitoring of inhibition due to matrix components. Although determining whether berries are contaminated requires the optimisation of various steps, from sample choice through to detection, the focus of our work here was towards the final detection step.

## Materials and Methods

### Sample Preparation

#### *C. cayetanensis* DNA

Purified DNA isolated from *C. cayetanensis* oocysts was kindly provided by Dr. Ynes Ortega, University of Georgia, United States. The DNA was isolated from positive faecal samples from Peru as previously described (Chandra et al., 2014). The DNA had a concentration of 32 ng/μl and this was serially diluted tenfold (3.2, 0.32, 0.032, and 0.0032 ng/μl) for the purpose of preparing the calibration curve. The concentration of the DNA was estimated from a spectrophotometric measurement of the purified qPCR product (NanoDrop ND-1000 Spectrophotometer, Saveen Werner AB).

Purified DNA of *C. cayetanensis* from four different sources (Guatemala, Malaysia, Israel, and unknown country of infection) were kindly provided by Dr. Jessica Beser, Public Health Agency of Sweden. These DNA isolates were used for testing the applicability of the new method for detecting isolates of *C. cayetanensis* from different geographic locations.

#### Oocysts of *C. cayetanensis*, *E. mitis, T. gondii, Cryptosporidium parvum*, and *Cystoisospora canis*

Unsporulated oocysts of *C. cayetanensis* in faeces were kindly provided by Dr. Kristin Elwin, Public Health Wales Health Protection Division, United Kingdom. The faecal sample was collected from a patient in Wales who had recently travelled to Mexico. The faecal sample containing the *Cyclospora* oocysts was washed twice with 0.5% SDS and the oocysts isolated using saturated salt flotation. The oocysts were suspended in distilled water and then stored in the refrigerator. These oocysts were used for evaluation of the performance characteristics of the developed method as applied on the berry matrix. Furthermore, the oocysts were sorted by fluorescence-activated cell sorting (BD FACSAria cell sorter), using their auto-fluorescence and size, into 96-well PCR plates at Ullevål Sykehus, Oslo, Norway.

Oocysts of *E. mitis* were isolated from chicken faeces, *C. canis* from canine faeces, and *C. parvum* from stool samples from calves. These samples had all been submitted for diagnostic analysis at the Parasitology Laboratory, Faculty of Veterinary Medicine, Norwegian University of Life Sciences. After repeated washing steps in water, the oocysts were isolated by saturated salt flotation and stored refrigerated. Oocysts of *T. gondii* from a previous project were also used; the details of the oocyst strain and origin are described elsewhere (Harito et al., 2016). The oocysts of *T. gondii* that had been stored in 2% H_2_SO_4_ were washed with water three times before proceeding to DNA extraction.

The number of oocysts from all parasites were estimated using KOVA^®^ Glasstic^®^ Slide 10 Microscope Slide (VWR, Norway).

#### Berry Matrices

Sample matrices were prepared from store-bought raspberries, blueberries, and strawberries as follows. About 30 g of berries was weighed into plastic boxes to which 200 ml of 0.1 or 1% Alconox^TM^ (Alconox, Inc., NY, United States) was added. The boxes were then placed on an automatic shaker (Heidolph Vibramax 100); raspberry samples were shaken at 300 rpm for 10 min, whereas blueberry and strawberry samples were shaken at 600 rpm for 10 min.

The eluate was then transferred into four 50 ml tubes for concentration by centrifugation at 1,690 rcf for 10 min and the supernatant removed by vacuum suction, leaving 10 ml of the sediment. The pooled sediment was centrifuged at 3,803 rcf for 10 min with a deceleration break set to 6 (on a scale of 0–9) and about 1.5 ml of the sediment was further concentrated down to 250 μl by centrifugation at 13,000 rcf for 5 min.

### Isolation of DNA

DNA was isolated from the oocysts of all five coccidian parasite species using the DNeasy PowerSoil Kit (Qiagen, Norway) following the manufacturer’s instructions with slight modifications. Briefly, 250 μl of the sample containing the parasites were subjected to bead-beating to break the oocyst walls and facilitate the release of DNA, using FastPrep-24 5G^TM^ High Speed Homogeniser (MP Biomedicals, France) in two cycles of 4 m/s for 60 s. The lysate was then centrifuged at 10,000 rcf for 1 min at room temperature, and 500 μl of the supernatant used for the subsequent step in the protocol. The effects of background DNA and PCR inhibitors from the sample matrix were tested by spiking the berries with *Cyclospora* oocysts and this was subjected to DNA extraction as described above. The final elution volume was 50 μl. Samples were stored at −20°C until further analysis.

### Real-Time PCR (qPCR) Assay

#### Primers and Probe Design

The primers and probe for *C. cayetanensis* were designed and tested using Geneious 11.1.4^3^ to amplify a product of 141 bp from a target region of the ITS-1 region, based on a consensus of nine sequences retrieved from the GenBank (GenBank Accession No. AF301386, AF301389, AF302506, AF302529, AF302533, AF302546, AF302558, GU295381, and GU295248). The oligos used in the present study are presented in Table 1.

**TABLE 1:** The overview of setup for the Duplex assay.

	***C. cayetanensis***	**Phocid herpesvirus 1 (PhHV-1)**
Forward primer (5′ → 3′)	CyITS1_TT-F ATGTTTTAGCATGTGGTGTGGC	GGGCGAATCACAGATTGAATC
Reverse primer (5′ → 3′)	CyITS1_TT-R GCAGCAACAACAACTCCTCATC	GCGGTTCCAAACGTACCAA
Probe (5′ → 3′)	CyITS1_TT-P HEX-TACATACCCGTCCCAACCCTCGA-BHQ1	6FAM-TTTTTATGTGTCCGCCACCATCTGGATC-BHQ1
Primers conc.	0.5 μM	0.2 μM
Probe conc.	0.15 μM	0.1 μM
Amplicon size	141 bp	89 bp
Thermal profile	95°C for 3 min 1 × 95°C for 15 s 45 × 60°C for 30 s 45 ×	
		

The primers and probe for detection of the internal control, Phocine herpesvirus-1(PhHV1), were as described previously (Niesters, 2002). Reverse-phase cartridge (RP1) purified primers and HPLC purified probes were purchased from Sigma-Aldrich.

#### qPCR Conditions

The PCR was performed in a 0.3 ml PCR plate without skirt (Multiply^®^, Sarstedt, Norway). The reaction conditions for the qPCR setup was a 20 μl reaction volume that included a template volume of 2 μl, 10 μl of 2 × KiCqStart^®^ Probe qPCR ReadyMix^TM^, low ROX^TM^ (Sigma-Aldrich, Norway), 0.5 μM of each primer and 0.1 μM of the probe. ROX was used as a reference dye against which the target fluorescence data were normalised.

### Method Evaluation

**FIGURE 1:**
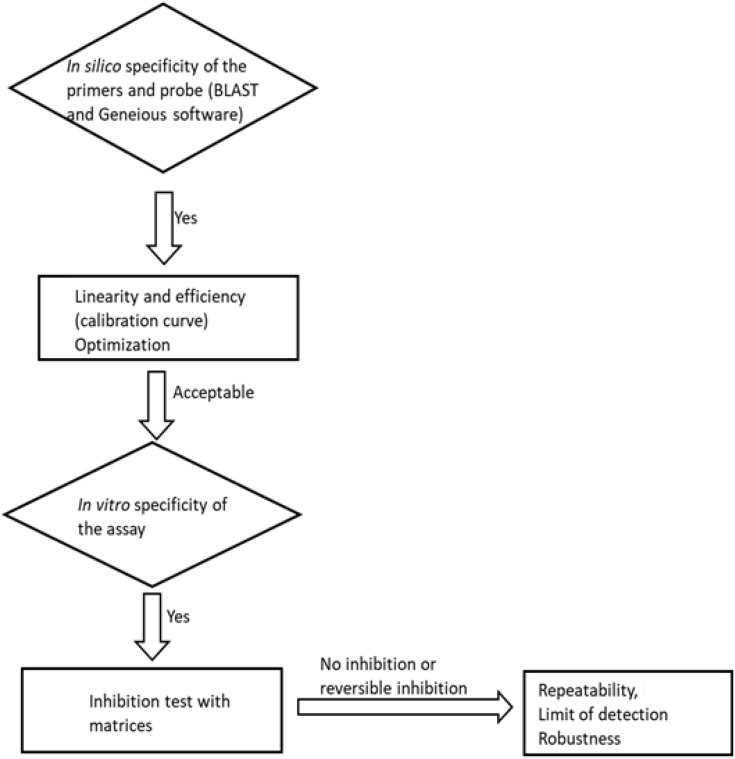
A flowchart of the method evaluation steps followed in this study.

The method was evaluated for its specificity, efficiency, linearity, inhibition, limit of detection (LoD), precision, and robustness. The planned steps for method evaluation are summarised in a flowchart (Figure 1).

#### Specificity

The specificity of the primers and probe were investigated *in silico* using BLAST searches against coccidia in general and separately for the *Cyclospora* genus. In order to increase the level of specificity, the “somewhat similar sequences (blastn)” algorithm was selected to allow cross-species comparison. The specificity was evaluated *in vitro* by running agarose gel electrophoresis (1.5%) of the qPCR product to confirm the amplicon size. In addition, DNA from related coccidia (*Toxoplasma, Eimeria, Cystoisospora*, and *Cryptosporidium*) were included in the qPCR run. Moreover, the qPCR product was sequenced by a commercial company (Eurofins Genomics, Germany GmbH) to confirm the specificity of the assay.

#### Efficiency and Linearity

The method was first evaluated for its efficiency and linearity for the range of concentrations used in this study (Section “Sample Preparation”). The calibration curve was prepared using tenfold serial dilutions of the pure *Cyclospora* DNA (64 ng, 6.4 ng, 0.64 ng, 64 pg, and 6.4 pg). The linearity of the method was assessed by obtaining the coefficient of determination, with *r*^2^ ≥ 0.98 considered acceptable.

#### Inhibition

Inhibition from the berry matrices was tested using a tenfold serial dilution of berry washes spiked with *Cyclospora* oocysts. To evaluate the applicability of the new method in outbreak investigations, where the samples may become old and deteriorated before reaching the laboratory, blueberries and raspberries kept in the fridge for 32 days were spiked with *Cyclospora* oocysts and processed for the qPCR detection.

#### Precision

The precision of each assay was estimated, under repeatability conditions, for three different berry matrices (raspberry, strawberry, and blueberry) containing approximately 0.16 and 3.2 ng of the target DNA and expressed as the standard deviation of C_q_ from 12 replicates of each.

#### Limit of Detection

The limit of detection was determined by dilution of the DNA and approximately 32, 12.8, and 6.4 pg of the DNA roughly estimated to be equivalent to 5, 2, and 1 oocyst based on the gene copy number, respectively, were tested. The qPCR was run with six replicates of the 32 pg and nine replicates of the 12.8 and 6.4 pg.

The LoD was also estimated using the flow-sorted *Cyclospora* oocysts (section “Oocysts of *C*. *cayetanensis, E*. *mitis, T*. *gondii, Cryptosporidium parvum*, and *Cystoisospora canis*”). The oocysts spiked into a tube containing the eluates of blueberry washes ready for DNA extraction. Accordingly, five replicates of the two oocysts, five oocysts, 10 oocysts, and 100 oocysts sorted by FACS were used. Based on the preliminary results from this experiment, 10 and 50 oocysts of *Cyclospora* (each in triplicate) were used for direct spiking on the berries before washing. In this experiment, about 20 μl of suspension containing the oocysts was used for the spiking and this was distributed to different individual berries. The spiked berries were left to dry at room temperature for 3 h and then stored in the refrigerator overnight. The berries were then subjected to washing as described in section “Berry Matrices”. The eluates were spiked to obtain an approximation of the LoD of the qPCR. Spiking of berries was conducted to assess the LoD of the entire method (including washing, DNA extraction, and detection with qPCR).

#### Robustness

The robustness of the assay was evaluated by introducing small, but deliberate, changes into various factors of the assay, including the commercially available master mixes, concentrations of primers and probe, annealing temperature, and volume of the super mix (containing all reagents except template). A screening experimental design that enables detection of the main effects was used for this experiment (Table 2). Nine replicates of *Cyclospora* DNA (approximately 1.28 ng) and a negative control were included per experimental setup (the six different combinations of the different factors).

**TABLE 2:** Experimental design for testing the robustness of the new assay.

**Factor**	**Combination**						**The new method**
	**Test-1**	**Test-2**	**Test-3**	**Test-4**	**Test-5**	**Test-6**	
Master mix type	−1	−1	−1	1	1	1	KicqStart
Primer conc.	1	1	−1	−1	−1	1	0.5 μm
Probe conc.	1	−1	1	−1	1	−1	100 nm
Super mix vol.	1	−1	−1	−1	1	1	18 μl
Annealing temp.	1	−1	1	−1	−1	1	60°C

**Sign used**	**Master mix type**			**Primer conc.**	**Probe conc.**	**Super mix vol.**	**Annealing temp.**

−1	KicqStart			0.4 μM	80 nM	17.1 μl	59°C
1	PerfeCTa Multiplex qPCR ToughMix			0.5 μM	100 nM	18.9 μl	61°C

Furthermore, considering the within species variation of ITS-1 copies, the newly developed method was tested on different isolates of *C. cayetanensis* DNA obtained from different sources. The sources included Guatemala, Malaysia, Israel, the United Kingdom, and one isolate for which the country of origin was not reported.

#### Duplexing With an Internal Control (PhHV-1)

In order to monitor success of DNA extraction as well as the PCR, the inclusion of PhHV-1 (EVAg Ref-SKU: 011V-00884) as an internal control was evaluated. The PhHV-1 sample (10 μl of the 1000-times diluted stock) was mixed with the *Cyclospora* oocysts samples for co-extraction of DNA. The specific details of the duplex assay are presented in Table 1.

The duplex assay was evaluated for the variation in the C_q_ value of the internal control by varying the concentration of *C. cayetanensis* DNA while keeping the concentration of PhHV-1 DNA constant. The DNA extracted from about 10^5^ oocysts was serially diluted to get approximately 10^4^, 10^3^, 10^2^, and 10 oocysts. The PCR was run in 20 μl volume with a 3.5 μl template, with 2 μl of templates added from the DNA extracted from the *C. cayetanensis* oocysts and 1.5 μl of PhHV-1 DNA was added to each well of the PCR plates.

Similarly, the effect of the internal control on the low concentration of *Cyclospora* DNA was also assessed by simultaneously running the singlex and duplex assays on the same serially diluted templates.

### Data Collection and Analysis

The fluorescence data were collected by Stratagene Mx3005P. The raw fluorescence intensity was evaluated against the recommended range of the instrument by using the multicomponent view. Each analysis was run in triplicate unless otherwise stated, and the mean C_q_ was used for calculations. The data obtained with the Mx3005P were then exported to an Excel sheet (Microsoft^®^ Office Excel^®^ 2010) for further statistical analysis by JMP^®^ Pro version 14.1.0 software (SAS institute, Inc.). The SD of C_q_ was calculated and presented using its 95% confidence interval. The efficiency of the qPCR was calculated automatically by the MxPro^TM^ qPCR software.

### Quality Control

An unspiked berry control was included in the experimental setup to ensure that the amplifications are specific to the *Cyclospora*. Every qPCR run was performed in triplicate and no template control (NTC) was included in every run. The TaqMan probes were prepared in small volumes of working solution to reduce the potential for damage from repeated freeze-thawing. Furthermore, to ensure that the fluorescence obtained was only from amplification of the template, no amplification control (NAC) was run after repeated freeze-thaws of the probe.

## Results and Discussion

The results of experiment conducted to select an appropriate concentration of Alconox for the washing of blueberries indicated that there was no meaningful difference in C_q_ values for the two concentrations (data not shown).

### Method Evaluation Using *C. cayetanensis* DNA

#### Specificity: *In silico* Test

The specificity test using BLAST search indicated that for both primers there was no hits for any other coccidia other than *C. cayetanensis*. The “blastn” search for the probe returned, in addition to *C. cayetanensis*, four hits for *Neospora caninum* complete genome (GenBank Accession No. LN714484.1, LN714488.1, FR823386.1, FR823385.1), and one hit for *Hammondia hammondi* phospholipid-translocating P-type ATPase (GenBank Accession No. XM_008885584.1). However, these alignments had higher E values (8), which indicates that the probability of the alignment to be just by chance is very high. Furthermore, the *Neospora* and *Hammondia* sequences were imported to the Geneious software to test the primers and probe for their potential to amplify and detect these unintended targets. The analyses showed no cross-reactivity with *H. hammondi* and *in silico* testing against the sequences of *N. caninum* identified no match with the set of primers and probe. However, it should be noted that there were many bases consecutively represented by “N” (which represents any of the four bases, A, G, T, or C of DNA) in this whole genome sequence of *N. caninum*.

#### Efficiency and Linearity

**FIGURE 2:**
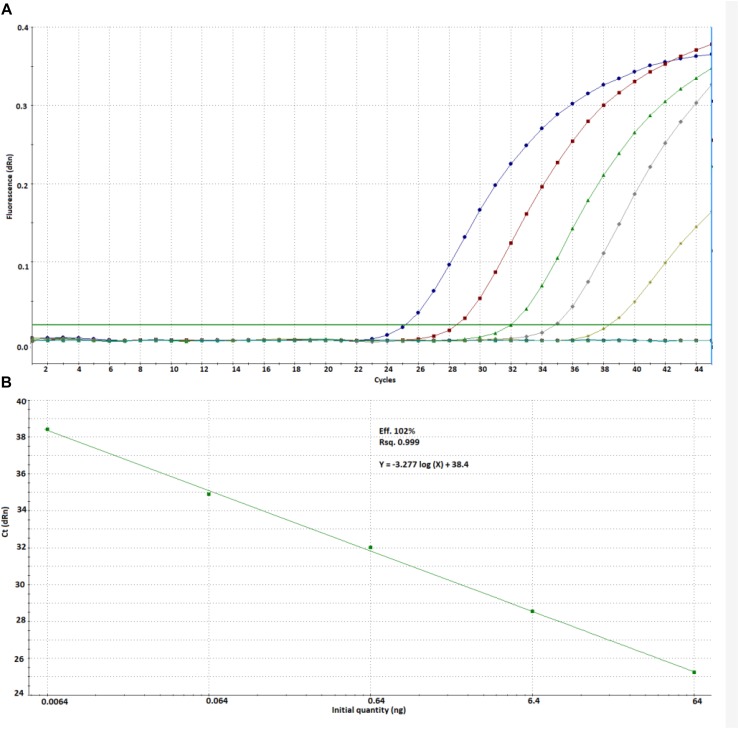
Amplification plot **(A)** and calibration curve **(B)** prepared from *Cyclospora* DNA by using the new method.

We found an ideal efficiency (102%) and linearity (*r*^2^ = 0.999) for the tested concentration range at a threshold fluorescence of 0.02 (Figure 2).

#### Specificity: *In vitro* Test

As the *in silico* test cannot entirely replace the practical test of specificity in the laboratory, the assay was evaluated for cross-reactivity against four genera of related coccidian parasites (*Cryptosporidium*, *Eimeria, Cystoisospora*, and *Toxoplasma*). Such empirical testing for *Hammondia* and *Neospora* was not conducted due to the lack of availability of these parasites. However, the *in silico* testing provided confidence that cross-reactivity would be highly unlikely to occur. No cross-reactivity between the related coccidian parasites tested, and the primers and probe used in the present assay was detected. The sequencing result also confirmed that the qPCR product was indeed from the amplification of the intended target in the ITS-1 region of *C. cayetanensis* based on the BLAST search of the sequences obtained. The BLAST search result showed 100% identity with many (at least 100) of the sequences of *C. cayetanensis* ITS-1 found in the GenBank (AF302599.1, AF302508.1, GU295401- GU295404, GU295365 - GU295371, to mention a few) with 100% query cover.

#### Inhibition Test

It is well known that berry fruits contain PCR inhibitors such as polysaccharides (e.g., pectin) and polyphenols (Schrader et al., 2012). It is therefore important that every assay designed to detect parasite contamination of this fresh produce type should determine the magnitude of inhibition from these matrices. In the present method, we detected no inhibition from the berry matrix. The applicability of the new method for outbreak investigation, as assessed using old berries spiked with *Cyclospora* oocysts, gave positive results with no inhibition from the matrix. This was confirmed by including the two-fold and four-fold diluted templates in the duplex qPCR run. Here it is worth noting that the amount of debris after the final concentration was more than twice than that obtained with fresh berries. However, the volume of the debris did not affect the DNA isolation, and there was no requirement to divide the sample into multiple tubes. Nevertheless, for samples resulting in a much larger sediment than required (250 μl) it would be possible to divide the sample between two tubes, and to then combine it into one spin column during the DNA binding step to avoid the risk of dilution.

#### Limit of Detection

The LoD was shown to be approximately 6.4 pg, with a probability of about 77% (seven positives out of nine replicates).

#### Precision: Repeatability

The repeatability of the assay was demonstrated by the minimal difference between replicates of the run, as shown in Table 3. At a lower concentration of template (160 pg), the overall repeatability with the berry matrices showed a C_q_ with SD of 0.4 (95% CI: 0.3, 0.5). The SD of C_q_ for raspberry was 0.5 (95% CI: 0.3, 0.8) and showed the highest deviation. For blueberry and strawberry, the SD was 0.3 (95% CI: 0.2, 0.5).

**TABLE 3:** Repeatability study of the new method for the three berry matrices spiked with *Cyclospora* DNA.

	**Mean C_q_ ± SD**	**95% CI**	
**0.160 ng DNA**			
Raspberry (*n* = 11)	35.07 ± 0.51	34.73 ± 0.36	35.41 ± 0.90
Strawberry (*n* = 12)	35.18 ± 0.30	35.00 ± 0.21	35.37 ± 0.50
Blueberry (*n* = 12)	34.74 ± 0.29	34.56 ± 0.20	34.92 ± 0.49
**3.2 ng DNA**			
Raspberry (*n* = 12)	30.71 ± 0.17	30.60 ± 0.12	30.82 ± 0.29
Strawberry (*n* = 12)	31.16 ± 0.22	31.02 ± 0.16	31.30 ± 0.37
Blueberry (*n* = 12)	30.98 ± 0.18	30.87 ± 0.13	31.09 ± 0.30

The repeatability was also evaluated for a higher concentration of template (3.2 ng). The results of the experiment showed a better precision, with an overall SD of 0.2 (95% CI: 0.2, 0.3) and the SDs for each matrix were similar.

#### Robustness Test

**FIGURE 3:**
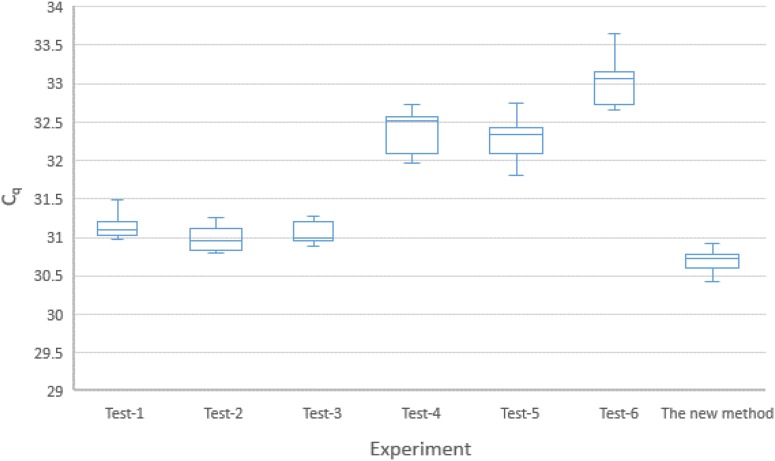
Box-plot (with 95% CI) representation of the six different experiments on the combinations of the five factors included in the robustness test (see Table 2). KicqStart Master Mix: Tests 1, 2, 3; PerfeCTa master mix: Tests 4, 5, 6. Primer concentration of 0.4 μM: Tests 3, 4, 5; Primer concentration of 0.5 μM: Tests 1, 2, 6. Probe concentration of 80 nM: Tests 2, 4, 6; Probe concentration of 100 nm: Tests 1, 3, 5. Super mix volume of 17.1 μl: Tests 2, 3, 4; Super mix volume of 17.1 μl: Tests 1, 5, 6. Annealing temperature at 59°C: Tests 2, 4, 5; annealing temperature at 61°C: Tests 1, 3, 6.

The findings of the experiments on robustness of the new method identified factors (as described in the sub-section on Robustness, in the section on Method Evaluation.) that could significantly affect the qPCR results. A visual summary of the experiment is presented by the Box–Whisker plot in Figure 3. The overall standard deviation of the C_q_ for the six combinations was 0.8 (95% CI: 0.7, 1.0). The coefficient of variation (CV) of the copy numbers was estimated to be 50% (95% CI: 48%, 54%). These figures might indicate that the new method was not sufficiently robust.

**FIGURE 4:**
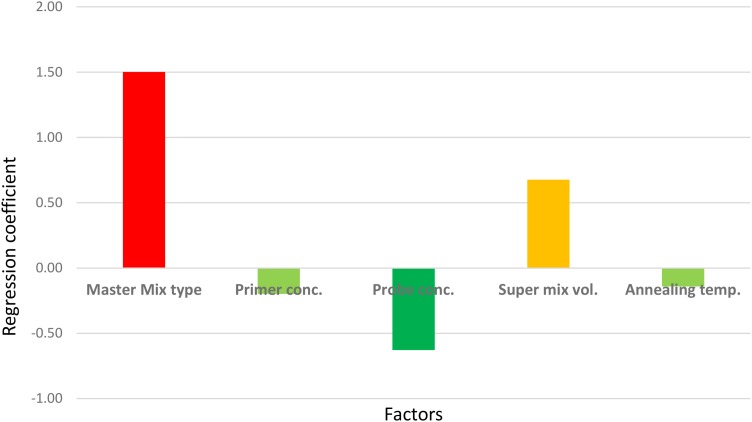
Screening for the main effects of factors that could affect the qPCR results. The sign (+ or –) of the coefficients indicates the direction of the factor’s effect on the C_q_ when changed from –1 to 1 (see Table 2).

However, the experiment clearly indicated which factor(s) most contributed to the large CV. As shown in Figure 4, the master mix type, the concentration of probe, and the volume of “super mix” contributed most to the deviations obtained. Changing the master mix type and using excess “super mix” volume resulted in higher C_q_ (the large and positive coefficients indicate increase in the C_q_). Nevertheless, maintaining the concentration of the probe as described in the protocol had a positive effect, indicated by the negative coefficient in the figure. There were no significant effects from either primer concentration or annealing temperature. The largest proportion of variation was due to the change in master mix type. In a separate run, in which all other assay conditions were kept constant except the master mix type, a difference of one cycle was noted (data not shown), which was also shown as the major contributor to the large CV in the robustness test results. Therefore, it should be noted that the performance of commercially available master mixes differ and should be considered during interpretation of qPCR results.

These results highlight the fact that in order for a new method to be applied in different laboratories, it should be sufficiently robust to be unaffected by small changes in the various factors that potentially impact on the performance of the assay. Conducting a robustness test is very helpful in predicting the outcome of inter-laboratory validation and enables necessary adjustments to be made to a protocol before investing a great deal of time and resources on inter-laboratory comparisons.

### Evaluation of the Method Based on Experiments With *C. cayetanensis* Oocysts

Considering the intergenomic and intragenomic variation of ITS-1 region, it might be beneficial to test different oocysts from various geographical locations. But one challenge is that there is a limited access to the oocysts of *C. cayetanensis* as humans are the only host. Oocysts of *C. cayetanensis* were obtained after completion of the development and evaluation of the method using the *C. cayetanensis* DNA.

With the use of the DNA extracted from the oocysts, the method showed a very good efficiency (92%) and linearity (0.999) (Supplementary Figure S1).

#### Duplexing With an Internal Control (PhHV-1)

The results indicated that the internal control could be included in a duplex assay for the detection of *C. cayetanensis* from berries without affecting the performance of the protocol. The assay showed a good efficiency (100 and 99.5%) and linearity (*r*^2^ = 0.99 and 1.00) for *C. cayetanensis* and PhHV-1, respectively (Supplementary Figure S2).

**FIGURE 5:**
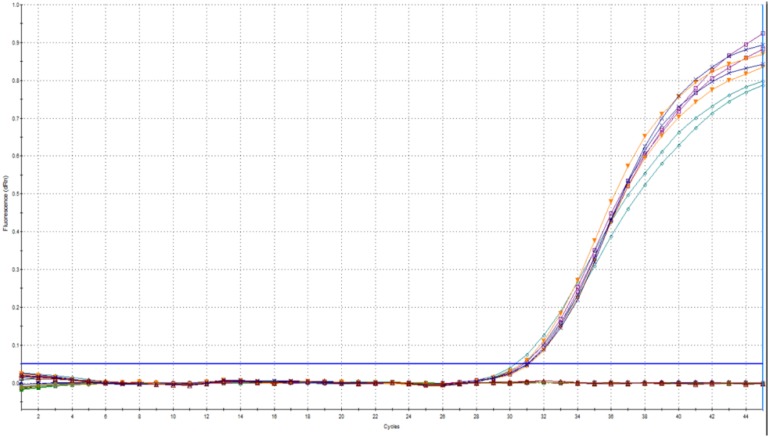
Different concentrations of *C. cayetanensis* (10^5^, 10^4^, 10^3^, 10^2^, and 10 oocysts) did not affect the C_q_ value of PhHV-1.

Furthermore, the test for the reliability of the internal control in a range of different concentrations of *C. cayetanensis* DNA showed that there was no significant variation in the C_q_ value (Figure 5). The standard deviation of the C_q_ for PhHV-1 was 0.2 (95% CI: 0.16, 0.49).

This indicated that PhHV-1 could be reliably used for monitoring inhibition due to matrix components. Adding the internal control to the sample before DNA extraction serves a dual role, monitoring both the presence of inhibition and the efficiency of the DNA extraction. This is best achieved by including a diluted template in the qPCR run. In cases where both the diluted and undiluted template results are negative for the internal control, failure of the DNA extraction is indicated. This is different from the approach used by Murphy et al. (2017) in which the internal control monitors the presence of inhibition only. However, it should be noted that a positive internal control might not be indicative of perfect DNA extraction because the efficiency of DNA extraction would not be the same for PhHV-1 as for the robust oocysts of *Cyclospora*. Nevertheless, this control would be useful to detect a major and inadvertent decline in the efficiency of the DNA extraction.

The internal control had no effect on detection of a low concentration of *Cyclospora* DNA (Table 4).

**TABLE 4:** The singlex and duplex qPCR results on the serially diluted oocysts of *Cyclospora* but with equivalent quantity of PhHV-1.

**Estimated no. of oocysts**	**C_q_ value obtained**	
	**Singlex assay**	**Duplex assay**
10^4^ oocysts	28.3	27.7
10^3^ oocysts	31.9	30.8
10^2^ oocysts	34.7	33.6
10 oocysts	37.9	37.6
No template control	No C_q_	No C_q_

Here it is also worth mentioning that the C_q_ value of the target is dependent on the matrix used during DNA extraction. With the use of DNeasy PowerSoil kit for the DNA isolation, it was noted that the C_q_ value of the target parasites were higher when purified samples were used. But using background matrices, such as berry washes, during the DNA extraction apparently reduced the C_q_ values. This was true for the qPCR tests on *E. mitis*, *T. gondii*, *C. cayetanensis*, and PhHV-1 (data not shown). In this study, the berry matrix seemed to improve the efficiency of the DNA extraction rather than reducing it. It is assumed that this enhanced DNA extraction may be due to a carrier function of one or more components in the berry matrix, but this was not further explored.

The duplex assay was also tested for its specificity, as was done for the singlex assay, and the result showed no C_q_ value for the related coccidian parasites (*Eimeria, Cryptosporidium, Toxoplasma*, and *Cystoisospora*). In every qPCR run, the NTC was included to rule out non-specific amplification. Furthermore, gel electrophoresis of the qPCR product of the duplex assay confirmed that there was no unintended amplification (Supplementary Figure S3).

#### LoD of the Duplex Assay

The findings of the present study showed that five oocysts of *Cyclospora* as the LoD of the duplex assay (Table 5).

**TABLE 5:** qPCR results of the eluates of blueberry washes spiked with 2, 5, 10, and 100 *Cyclospora* oocysts.

**Independent replicates**	**qPCR result**			
	**2 oocysts**	**5 oocysts**	**10 oocysts**	**100 oocysts**
1	No C_q_	Pos.	No C_q_	Pos.
2	No C_q_	Pos.	Pos.	Pos.
3	No C_q_	No C_q_	Pos.	Pos.
4	No C_q_	Pos.	Pos.	Pos.
5	No C_q_	No C_q_	Pos.	Nd

*Key: Nd – not done; Pos. – positive.*

Other studies on the detection of *C. cayetanensis* from fresh produce have reported different detection limits. A PCR method that could detect 40 oocysts per 100 g of raspberries has been reported (Steele et al., 2003). Lalonde and Gajadhar (2008) reported a PCR method that could detect a single oocyst from basil wash. However, the LoD of these methods are not strictly comparable because of the differences in the sample matrix and protocols for washing the berries.

It should be noted that the LoD could differ due to the sporulation status of the oocysts used in the experiments. This is because the number of gene copies would increase as the oocyst sporulates. In this study none of the oocysts were sporulated. It is also worth noting that the aim of the study should be considered for comparison of the LoD of methods. For studies intending to assess the sensitivity of the PCR, using a synthetic positive control gene target (Murphy et al., 2017) would be appropriate. However, this should not be confused with the LoD of the whole method that includes all the steps of analysis; washing, concentration, extraction of DNA, and PCR.

The results from berries spiked with 10 and 50 oocysts of *Cyclospora* and subjected to washing and DNA isolation for the detection with the duplex qPCR showed that it is possible to detect 10 oocysts of *Cyclospora* from 30 g of raspberries and blueberries (Table 6).

**TABLE 6:** The duplex qPCR results of the berries spiked with 10, and 50 oocysts of *Cyclospora*.

**Types of berries**	**qPCR result**		
	**Negative control (0 oocyst)**	**10 oocysts**	**50 oocysts**
**Raspberry**			
1	No C_q_	Pos.	Pos.
2	No C_q_	Pos.	Pos.
3	Nd	Pos.	Pos.
**Blueberry**			
1	No C_q_	Pos.	Pos.
2	No C_q_	Pos.	Pos.
3	Nd	No C_q_	Pos.

Here we have shown that this protocol is able to detect as few as ten unsporulated *Cyclospora* oocysts from 30 g of berries. However, as the ploidy of a sporulated oocyst is presumably 4 times higher than that of an unsporulated oocyst, we might speculate that for sporulated oocysts, or for mixed populations of sporulated and unsporulated oocysts, the LoD could be even lower. Due to the lack of availability of sporulated oocysts we were unable to test this empirically.

To maximise the sensitivity of the method, use of larger volumes of template (e.g., 5 μl) is recommended, and also replicates of the sample (at least triplicate). The template volume depends on whether the template contains inhibitors and this, in turn, depends on the efficacy of the DNA isolation kit at removing potential inhibitors. Thus, the practicality of using a larger template volume should be assessed in the laboratory. In the present study, the use of 2, 3, and 5 μl of the template was tested and lower C_q_ values were obtained with the higher template volume, which indicates that there was no inhibition due to the matrix components.

## Limitations

We assumed that the most appropriate DNA extraction protocol for *Cyclospora* oocysts would be similar to that for *T. gondii* oocysts, taking in to account their genetic and morphological similarities. In our laboratory, it has been shown that different commercially available DNA extraction kits have highly significant differences in their efficacy at extracting DNA from *T. gondii* oocysts (unpublished data). This might be due to differences in the lysis buffers in the kits or inhibition of qPCR due to chemicals used in the kits. Therefore, future standardisation of the present assay should include testing the efficiency of the DNA extraction protocol on oocysts of *C. cayetanensis*.

Furthermore, the high variability of the ITS-1 region of *C. cayetanensis* genome might affect the PCR. It was noted that the probe has up to three mismatches with some of the sequences of *C. cayetanensis* that have been submitted to GenBank. Although high variability at a target gene might be advantageous for source tracking and some epidemiological studies, it might also mean that it is challenging to design a primer pair and probe that is appropriate for amplification of DNA from all *C. cayetanensis* isolates. Nevertheless, it is worth noting that the ITS-1 variation reported did not show geographic cluster, but the variation was between and within samples examined (Olivier et al., 2001). In this study, *Cyclospora* isolates from six different sources were successfully amplified with the newly developed duplex qPCR method; although it would obviously be preferable to use further isolates. Thus, these data suggest that this detection method could be a suitable alternative for use in the analyses of berry samples and other fresh produce for *Cyclospora* contamination. It should be noted that in implementing new laboratory protocols, sequencing is an option for providing confidence regarding positive results.

In the present study, we have attempted to investigate the robustness of the new method by manipulating factors such as the annealing temperature, super mix volume, master mix brands, the concentration of primers and probes. However, there are also factors that could affect the robustness of the assay that has not been considered in our study. These include factors such as the analyst performing the test and the qPCR instrument brand. Such factors would be most appropriate to address by inter-laboratory comparison studies.

## Conclusion

In this study, a new assay targeting the ITS-1 target was developed for detection of *C. cayetanensis* as contaminants of berries and shown to be an effective approach that could be suitable alternative for food testing laboratories. The high specificity of the new detection method, as shown by both *in silico* and *in vitro* investigations, is a very important and relevant aspect. In addition to the specificity, the new protocol is robust (can tolerate minor changes in the annealing temperature, primers and probe concentration, and the reaction volume) and relatively simple, which makes it convenient for regular use in food testing laboratories. However, in order to standardise this method, further tests are warranted. This should include an inter-laboratory comparison for validation of the method’s fitness for purpose. Furthermore, any changes in the method should be assessed accordingly.

## Data Availability

All datasets generated for this study are included in the manuscript and the Supplementary Files.

## Author Contributions

KT and LR conceived and supervised the study, wrote the grant proposal, obtained the funding, and contributed to the design of experiments. TT designed and performed the experiments, analysed the data, and drafted the manuscript. All authors revised the manuscript and read and approved the final version of the manuscript.

## Conflict of Interest Statement

The authors declare that the research was conducted in the absence of any commercial or financial relationships that could be construed as a potential conflict of interest.

## Footnotes

1 https://www.cdc.gov/parasites/cyclosporiasis/outbreaks/index.html

2 https://eu.idtdna.com/pages/education/decoded/article/two-quenchers-are-better-than-one

3 https://www.geneious.com

## References

[B1] Almeria S. da Silva A. J. Blessington T. Cloyd T. C. Cinar H. N. Durigan M. (2018). Evaluation of the U.S. food and drug administration validated method for detection of *Cyclospora cayetanensis* in high-risk fresh produce matrices and a method modification for a prepared dish. *Food Microbiol.* 76 497–503. 10.1016/j.fm.2018.07.013 30166179

[B2] Casillas S. Bennett C. Straily A. (2018). Notes from the field: multiple cyclosporiasis outbreaks-United States, 2018. *MMWR Morb. Mortal Wkly. Rep.* 67 1101–1102. 10.15585/mmwr.mm6739a6 30286055 PMC6171894

[B3] Chacin-Bonilla L. (2017). “*Cyclospora cayetanensis*,” in *Global Water Pathogens Project*, eds Fayer R. Jakubowski W., (Lansing, MI: Michigan State University).

[B4] Chandra V. Torres M. Ortega Y. R. (2014). Efficacy of wash solutions in recovering *Cyclospora cayetanensis*, *Cryptosporidium parvum*, and *Toxoplasma gondii* from basil. *J. Food Prot.* 77 1348–1354. 10.4315/0362-028X.JFP-13-381 25198596

[B5] Harito J. B. Campbell A. T. Prestrud K. W. Dubey J. P. Robertson L. J. (2016). Surface binding properties of aged and fresh (recently excreted) *Toxoplasma gondii* oocysts. *Exp. Parasitol.* 165 88–94. 10.1016/j.exppara.2016.03.022 27003461

[B6] Lainson R. (2005). The genus *Cyclospora* (Apicomplexa: Eimeriidae), with a description of *Cyclospora schneideri* n.sp. in the snake *Anilius scytale* (Aniliidae) from Amazonian Brazil: a review. *Memórias do Instituto Oswaldo Cruz* 100 103–110. 10.1590/s0074-02762005000200001 16021295

[B7] Lalonde L. F. Gajadhar A. A. (2008). Highly sensitive and specific PCR assay for reliable detection of *Cyclospora cayetanensis* oocysts. *Appl. Environ. Microbiol.* 74 4354–4358. 10.1128/AEM.00032-08 18502915 PMC2493149

[B8] Murphy H. R. Cinar H. N. Gopinath G. Noe K. E. Chatman L. D. Miranda N. E. (2018). Interlaboratory validation of an improved method for detection of *Cyclospora cayetanensis* in produce using a real-time PCR assay. *Food Microbiol.* 69 170–178. 10.1016/j.fm.2017.08.008 28941898

[B9] Murphy H. R. Lee S. da Silva A. J. (2017). Evaluation of an improved U.S. Food and drug administration method for the detection of *Cyclospora cayetanensis* in produce using real-time PCR. *J. Food Prot.* 80 1133–1144. 10.4315/0362-028X.JFP-16-492 28590822

[B10] NiestersH. G. M. (2002). Clinical virology in real time. *J. Clin. Virol.* 25 3–12. 10.1016/S1386-6532(02)00197-X12467772

[B11] Olivier C. van de Pas S. Lepp P. W. Yoder K. Relman D. A. (2001). Sequence variability in the first internal transcribed spacer region within and among *Cyclospora* species is consistent with polyparasitism. *Int. J. Parasitol.* 31 1475–1487. 10.1016/S0020-7519(01)00283-1 11595235

[B12] Riner D. K. Nichols T. Lucas S. Y. Mullin A. S. Cross J. H. Lindquist H. D. A. (2010). Intragenomic sequence variation of the ITS-1 region within a single flow-cytometry–counted *Cyclospora cayetanensis* Oocysts. *J. Parasitol.* 96 914–919. 10.1645/GE-2505.1 20950098

[B13] Schrader C. Schielke A. Ellerbroek L. Johne R. (2012). PCR inhibitors – occurrence, properties and removal. *J. Appl. Microbiol.* 113 1014–1026. 10.1111/j.1365-2672.2012.05384.x 22747964

[B14] Shields J. M. Joo J. Kim R. Murphy H. R. (2013). Assessment of three commercial DNA extraction kits and a laboratory-developed method for detecting *Cryptosporidium* and *Cyclospora* in raspberry wash, basil wash and pesto. *J. Microbiol. Methods* 92 51–58. 10.1016/j.mimet.2012.11.001 23147278

[B15] Steele M. Unger S. Odumeru J. (2003). Sensitivity of PCR detection of *Cyclospora cayetanensis* in raspberries, basil, and mesclun lettuce. *J. Microbiol. Methods* 54 277–280. 10.1016/s0167-7012(03)00036-8 12782383

[B16] Verweij J. J. Laeijendecker D. Brienen E. A. T. van Lieshout L. Polderman A. M. (2003). Detection of *Cyclospora cayetanensis* in travellers returning from the tropics and subtropics using microscopy and real-time PCR. *Int. J. Med. Microbiol.* 293 199–202. 10.1078/1438-4221-52 12868656

