# Supplementary material for: A New Protocol for Molecular Detection of Cyclospora cayetanensis as Contaminants of Berry Fruits
Source: Front Microbiol. 2019 Aug 27;10:1939. doi: 10.3389/fmicb.2019.01939 (PMC6719520; doi:10.3389/fmicb.2019.01939)
Supplement: Supplementary file 1 [file Data_Sheet_1.docx]

***Supplementary Materials***

Supplementary Figure 1.


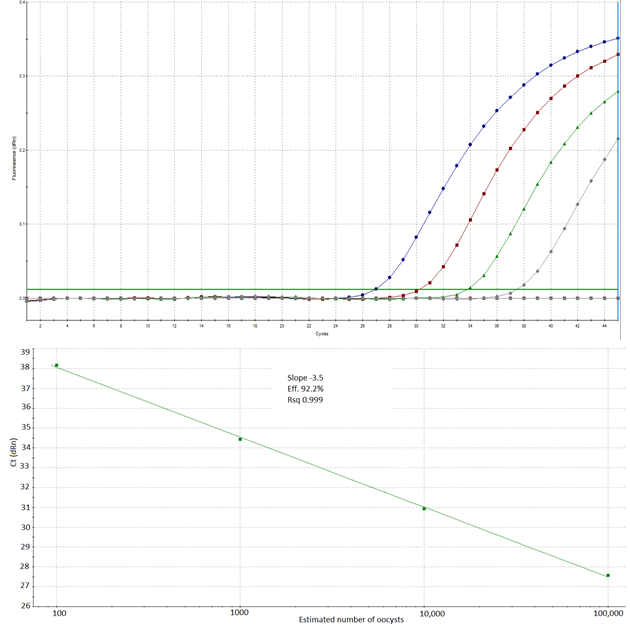


Supplementary Figure 1. The amplification plot (top) and standard curve (bottom) prepared by using the DNA extracted from the oocysts of *C. cayetanensis*.

Supplementary Figure 2.


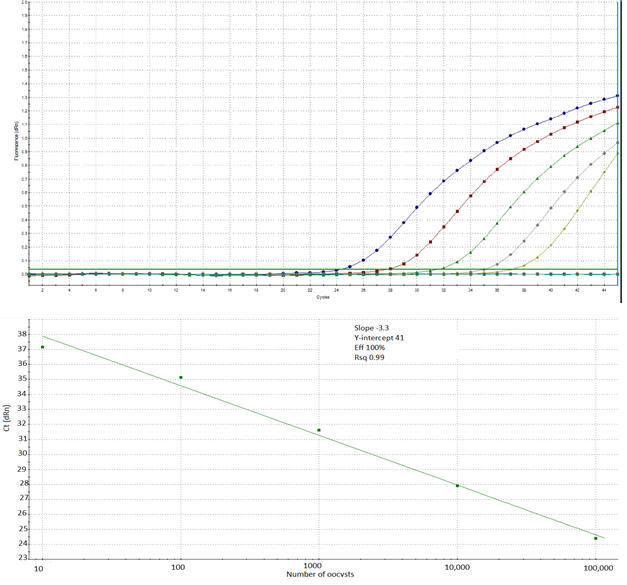


Supplementary Figure 2. Amplification plot (top) and standard curve (bottom) of *C. cayetanensis* target by using the Duplex assay

Supplementary Figure 3.


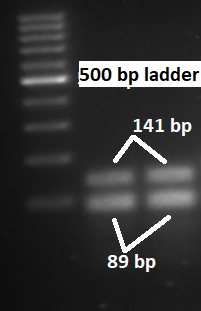


Supplementary Figure 3. Gel-electrophoresis of the PCR product of the duplex assay
